# Effects of environmental enrichment upon ethanol-induced conditioned place preference and pre-frontal BDNF levels in adolescent and adult mice

**DOI:** 10.1038/s41598-017-08795-0

**Published:** 2017-08-17

**Authors:** Ricardo Marcos Pautassi, Andrea B. Suárez, Lucas Barbosa Hoffmann, André Veloso Rueda, Mariana Rae, Priscila Marianno, Rosana Camarini

**Affiliations:** 1 Instituto de Investigación Médica M. y M. Ferreyra (INIMEC–CONICET-UNC), Córdoba, Argentina; 2 Departamento de Farmacologia, Instituto de Ciências Biomédicas, Universidade de São Paulo, São Paulo, SP Brazil; 3 Facultad de Psicología, Universidad Nacional de Córdoba, Córdoba, C.P. 5000 Argentina; 4 Laboratorio de Psicología Experimentaly Aplicada, IDIM, CONICET, UBA, Buenos Aires, Argentina

## Abstract

Environmental enrichment (EE) provides a non-pharmacological tool to alter drug-induced reward, yet its effects on ethanol-induced reward remain controversial. We analyzed adolescent vs. adult (mice) differences in the influence of EE on ethanol-induced conditioned place preference (CPP). The effects of these treatments on brain-derived neurotrophic factor (BDNF) levels in the prefrontal cortex were examined in a separate group of animals. Ethanol-induced CPP was found in adults, and it was similar in EE and in animals reared under standard housing conditions (SC). Adolescents kept under EE, but not those in SC, exhibited CPP. Among SC, but not among EE, adolescents, BDNF levels were significantly lower in those treated with ethanol than in those given vehicle. These results indicate that, compared to adults, adolescent exhibited reduced sensitivity to ethanol’s rewarding effects, yet the youth but not the adults exhibited sensitivity to the promoting effect of EE upon CPP by ethanol. Ethanol significantly reduced BDNF levels in adolescents reared under standard housing conditions, but not in adult mice nor in adolescents given EE housing conditions. The present results add to the plethora of adolescent-specific responses to ethanol or to environmental stimuli that may put the youth at risk for escalation of ethanol intake.

## Introduction

Sensitivity to the appetitive effects of ethanol predicts predisposition to ethanol intake. Greater ethanol-induced stimulation has been found in subjects at risk for ethanol dependence than in control subjects^1^, and mice displayed higher ethanol consumption after developing ethanol-induced sensitization compared with non-sensitized controls^2^. Age-related differences in sensitivity to the appetitive effects of ethanol have often been observed. Adolescent mice drink larger quantities of ethanol than adults and exhibit both less conditioned aversion induced by ethanol^3^ and greater ethanol-induced motor activity^4^ than adult counterparts. Adolescent mice have been shown to require a higher dose of ethanol, more conditioning trials^5^, an ethanol pre-exposure phase^6^, or training under stress conditions^7^ to express similar ethanol-mediated conditioned place preference (CPP) to adult mice. In CPP studies, the preference for a chamber that is paired with the effects of ethanol is considered an index of the appetitive effects of the drug. An experiment also showed that repeated treatment with 2.0 g/kg ethanol in a distinctive environment induced behavioral sensitization in adult but not adolescent mice^8^.

Environmental enrichment (EE) provides a non-pharmacological alternative to alter drug-induced reward. Rodents reared under EE conditions are exposed to several stimuli in their home cage, including toys, ladders, tunnels, and voluntary physical activity. Environmental enrichment decreases CPP induced by heroin^9^ and cocaine^10^, the self-administration of amphetamine^11^ and ethanol-induced behavioral sensitization^12^. EE has also been reported to reduce anxiety levels during exploration of the elevated plus maze and diminish the corticosterone response following exposure to novelty, effects that were exacerbated in rats that were selectively bred for high anxiety responses^13^. The effects of EE appear to be at least partially related to a facilitatory effect on cell proliferation and the number of dendritic spines and alterations in the levels of brain-derived neurotrophic factor (BDNF) in several areas, including the prefrontal cortex (PFC)^12^.

Unlike the consistent inhibition exerted by EE on cocaine- or amphetamine-induced reward, the effects of EE on ethanol-induced reward remain controversial. Initial studies^14–17^ indicated a facilitative effect of EE on ethanol intake. Similarly, a recent study^18^ reported greater ethanol intake and greater risk-taking behaviors in male rats that had been reared under EE throughout adolescence, when compared to control-housed counterparts. Other work, however, reported lower ethanol consumption and lower ethanol-induced CPP after EE in rats that were selected for high ethanol preference^19^ or spontaneous hypertension^20^. More recent studies demonstrated that EE decreased ethanol consumption after restraint stress^21^ and that EE blocked the development and expression of ethanol-induced behavioral sensitization in peri-adolescent Swiss mice^12^. EE also decreased BDNF levels in the PFC, but not in hippocampus. Repeated ethanol administration had an independent, reducing effect on BDNF levels^12^. A subsequent study gave mice biweekly ethanol injections and classified the animals based on their level of behavioral sensitization^22^. Compared with sensitized mice, non-sensitized mice exhibited decreases in BDNF and TrkB (the putative receptor activated by BDNF) mRNA levels in several brain areas, including the prelimbic PFC. It seems clear that more information is needed to understand the modulatory effects of EE on ethanol-mediated reward, the associated changes in BDNF levels at PFC, and age-related changes associated with these phenomena.

The present study analyzed age-related (i.e., adult *vs*. adolescent Swiss mice, Experiments 1 and 2, respectively) differences in the modulatory influence of EE on ethanol-induced CPP, and the effect of repeated ethanol treatment on BDNF levels in the PFC of adult and adolescent Swiss mice, exposed or not to EE (Experiment 3). PFC was selected based on its involvement in extinction of drug-induced motivational learning^23^. The hypotheses were that adolescents would exhibit differential sensitivity to CPP than adults. The direction of this difference was hard to predict. Rat studies^24–26^ have suggested greater CPP by ethanol in adolescent than in adults (with the latter age group typically exhibiting conditioned place aversion by ethanol), but some mice studies suggest the opposite^7, 27^. It was thus also possible to presume reduced CPP by ethanol in adolescent than in adult mice. A repeated testing protocol (i.e., four tests, spread across the week after conditioning) was used. Repeated, as opposed to single-session, testing increases the chance of detecting treatment-related differences in the expression of CPP. Indeed, a novelty in this investigation is the comparison of ethanol-induced CPP in adolescent and adult mice, using the same dose, apparatus and conditioning procedures and using a repeated testing protocol. Although comparisons between ethanol-induced CPP in adolescent and adult mice have been conducted^6^, patterns of expression of ethanol-induced CPP across repeated testing have yet to be compared at these ages. The information derived from the age comparison should help elucidate if the usual pattern of mice exhibiting reliable ethanol-mediated place conditioning is altered when testing takes places at adolescence rather than at adulthood. We also expected that EE animals would be less sensitive to the appetitive effects of ethanol and exhibit alterations in BDNF levels compared with animals that were reared under standard conditions.

## Methods and General Procedures

### Experimental designs

Experiments 1 (adolescents) and 2 (adults) used a 2 × 2 factorial design: environmental housing conditions before and during CPP procedures [environmental enrichment (EE) *vs*. standard housing conditions [SC]) × treatment during CPP (animals that received ethanol vs. animals that received only vehicle). Each group was composed of 9–10 animals. A 2 (age: adolescent *vs*. adult) × 2 (environmental condition before and during ethanol pre-exposure procedures: EE *vs*. SC) × 2 (treatment: ethanol or vehicle) factorial design was used in Experiment 3. The adult groups of Exp. 3 had 9 animals each, whereas adolescent groups had 8–12 subjects.

### Subjects

A total of 75 adolescent (37 and 38 in Experiments 1 and 3, respectively) and 80 adult (39 and 41 in Experiments 2 and 3, respectively) male Swiss mice were used. The animals were born in the vivarium of the Instituto de Ciências Biomédicas, Universidade de São Paulo (São Paulo, Brazil). The colony was kept at an ambient temperature of 21 °C, and the lights were turned on and off at 7:00 AM and 7:00 PM, respectively. The mice had free access to food and water. Rearing and experimental procedures were approved by the Ethical Committee for Animal Use of the Instituto de Ciências Biomédicas, Universidade de São Paulo. The experiments were also carried out in accordance with the Declaration of Helsinki and the Guide for the Care and Use of Laboratory Animals^28^ as adopted and promulgated by the NIH and the EU.

### Rearing procedures

**Figure 1:**
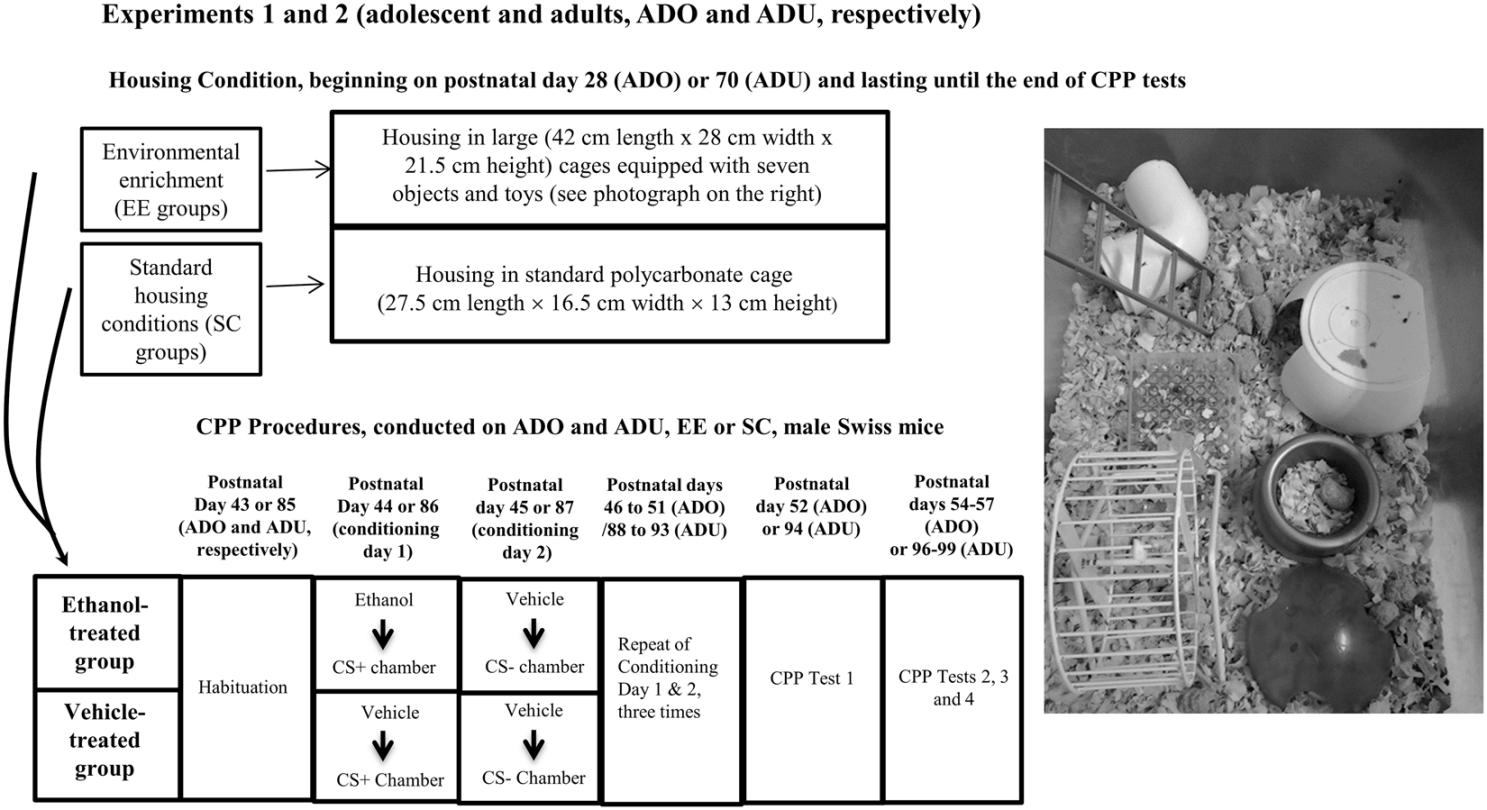
Adolescent and adult mice under environmental enrichment conditions were housed in large transparent polycarbonate cages (42 cm length × 28 cm width × 21.5 cm height) equipped with seven objects and toys, including a running wheel, ladders, pipes and house-like objects. One of the objects was a round container that contained food. The placement and composition of these non-chewable plastic objects were changed every week to prevent habituation. The photograph illustrates one of these compositions.

Adolescent and adult animals were housed five per cage, throughout the whole study, in polycarbonate cages lined with pine shaving. Differential rearing conditions began on postnatal day (PD) 28 for adolescents or PD70 for adults. The rationale for starting EE in adults at PD70, rather than starting it at PD28 and rearing them in the EE environment all through adulthood, was to equate the level of EE exposure across age. Assignment to EE or SC conditions was pseudorandom, as groups were balanced for body weight. On PD28 or PD70, the animals that were assigned to SC conditions were transferred to a new, standard polycarbonate cage (27.5 cm length × 16.5 cm width × 13 cm height), and mice in the EE groups were housed in large transparent polycarbonate cages (42 cm length × 28 cm width × 21.5 cm height) that were equipped with seven objects and toys, including ladders, pipes, house-like objects, and a running wheel (see Fig. 1). The placement and composition of these non-chewable plastic objects were changed every week to prevent habituation. The animals were kept under SC or EE conditions throughout the course of the experiment, as shown in the timeline of Fig. 1. They were identified using tail marks made of permanent marker and dots made of picric acid.

### Drugs

Ethanol (Merck do Brasil, Rio de Janeiro, RJ, Brazil) was intraperitoneally (i.p.) administered at a dose of 2.0 g/kg. This dose was achieved by administering 0.015 ml of an ethanol solution at 16.8% (v/v), per gram of body weight. Intraperitoneal injections were performed in less than 10 s between the diaphragm and genitalia. Control mice were administered isovolumetric injections of the vehicle solution (0.9% v/v saline).

### Conditioned place preference

The CPP apparatus employed in Experiments 1 and 2 was rectangular (44.5 cm × 14 cm × 14 cm) and had three compartments. The central area had a solid gray smooth floor (8.5 cm × 14 cm). The other two compartments (each 18 cm × 14 cm) were white but differed in the direction of black wall stripes and flooring. One had horizontal stripes and a stainless-steel mesh floor. The other had vertical stripes and a rod floor. Removable guillotine doors granted access to each compartment.

The CPP procedure began on PD43 or PD85 (adolescent and adult mice, respectively) after 15 days of SC or EE exposure and involved one habituation session, eight conditioning sessions, and four test sessions^29^, as described in the timeline of Fig. 1.

During the habituation session, the animals received a saline injection and then were gently placed in the central compartment of the apparatus for 15 min with free access to all of the compartments. The aim of this session was to familiarize the animals with the apparatus and provide a measure of preconditioning place preference.

Conditioning started 24 h following habituation and consisted of daily 5-min sessions, in which animals received either ethanol or saline and were then confined to only one of the conditioning compartments for 5 min. Animals were placed within the CPP chamber immediately after ethanol administration. Animals in the experimental group were given alternating injections of ethanol or saline. Half of these animals received ethanol in the rod floor compartment, and half of the animals received ethanol in the mesh floor compartment. Hereinafter, we refer to the compartments that were paired with ethanol and vehicle administration as CS^+^ and CS^−^, respectively. During the first conditioning day, half of these animals received ethanol, and the other half received saline. The duration of the conditioning trial (i.e., 5 min) was chosen because it is the standard length for the duration of exposure to the CS in ethanol studies in mice, as established in parametric studies by Cunningham and co-workers^29^. In a seminal study^30^ it was observed that the longer the duration of exposure to the CS the more attenuated the ethanol-induced CPP.

Control animals were given only saline across the eight sessions. For the statistical analysis, in these control animals one of the compartments was randomly assigned to be the CS^+^ and the other was designated as the CS^−^. Specifically, in half of the controls the CS^+^ was the rod floor compartment, whereas in the other half was the mesh floor compartment. In other words, an unbiased CPP design was employed. It is conceivable that the use of a biased CPP design (e.g., pairing ethanol with the less preferred compartment) would have helped avoid potential pre-conditioning chamber bias. We, however, deemed a biased CPP design not recommendable for the purpose of our study. Ethanol has, in addition to appetitive effects, anxiolytic or anti-anxiety effects^31^ that can endow the chamber with conditioned incentive properties. Had we paired ethanol with the less preferred chamber then we would have faced the caveat of not being able to tell if the CPP was attributable to the appetitive or to the anxiolytic effect of the drug, or to some combination of these effects [for a full discussion of the advantages and disadvantages of biased CPP designs, see ref. 32].

The first of the four tests was conducted a day after the last conditioning session. Three days after terminating conditioning, another test was conducted. The two additional test sessions (i.e., tests 3 and 4) took place 5 and 6 days after the end of conditioning procedures. During each test, the mice were withdrawn from the home cage and placed into the CPP apparatus. They could freely explore all of the compartments for 15 min.

Habituation and test sessions were registered via a video camera mounted on the ceiling. The video files were then analyzed by two researchers, blind to the experimental conditions of the mice, for time spent in the different sections of the apparatus. In the habituation session and in each test, the time spent in the rod or mesh floored compartments were recorded whenever the head and front paws were positioned over a section of the cage. The dependent variable for the tests sessions was the total or percent time (in seconds) spent in the CS^+^ compartment. In other words, CPP scores took into account the time spent in the compartment that was paired with ethanol’s post-absorptive effects, regardless of whether this was the mesh or rod compartment.

### Tissue collection and measurement of BDNF

In Experiment 3 the animals, adolescents and adults, underwent the same control or EE housing conditions and the same CPP training described in Experiments 1 and 2. This implied that half of the subjects in each age group received intermittent ethanol administration (4 every-other-day injections, dose: 2.0 g/kg) and half only saline injections, mimicking the CPP protocol. Instead of being tested for CPP on PD52 or PD94 (adolescents and adults, respectively) the subjects were submitted to a tissue collection procedure: they were euthanized (cervical dislocation) and the PFC was rapidly dissected, weighed, placed on dry ice, and stored at −80 °C. The samples were obtained and BDNF measurements were performed following the protocol described by Rueda, *et al*.^12^. Briefly, 1 ml of lysis buffer was added to each sample, followed by sonication for 15 s. Three milliliters of lysis buffer was subsequently added, followed by sonication for 15 s and centrifugation for 30 min at 16.000 RMP at 4 °C. The supernatants were stored at −80 °C. The BDNF Emax ImmunoAssay System (Promega, Madison, WI, USA) was used to detect BDNF levels. Each well of a 96-well microplate was covered with 100 ml anti-BDNF monoclonal antibody diluted 1:1000 in carbonate buffer (25 mM NaHCO_3_ and 25 mM Na_2_CO_3_, pH 9.7). The sealed plate was incubated overnight at 4 °C and then washed with Tris-buffered saline Tween (TBST) washing buffer (20 mM Trizma [pH 7.6; Sigma-Aldrich, St. Louis, MO, USA], 150 mM NaCl, and 0.05% Tween 20 [Labsynth, Diadema, SP, Brazil]), and 200 ml/well of 1x Block & Sample buffer was added.

Following 60 min incubation at room temperature, the plate was washed three times. Afterward, 100 ml of BDNF standard (serial 1:2 dilutions, ranging from 500 to 0 pg BDNF/ml) or the sample (1:4 dilution) were added to each well in duplicate. The plate was shaken at room temperature for 120 min and then washed five times with TBST wash buffer. We then added 100 ml per well of anti-BDNF polyclonal antibody diluted 1:500 in 1x Block & Sample buffer. The sealed plate was incubated at room temperature with shaking for 4 h and then washed five times, and 100 ml of anti-immunoglobulin Y horseradish peroxidase conjugate (diluted 1:200 in 1x Block & Sample buffer) was added to each well. The sealed plate was protected from light and incubated for 1 h with shaking and then washed five times. The final step was 10 min incubation with shaking at room temperature with 100 ml/well of TMB One solution. The reaction was stopped by adding 100 ml of 1 N HCl, and absorbance was measured at 450 nm. Values are expressed as pg/mg.

### Data analysis

Data from two adults were lost because of procedural errors during the CPP procedures, and a BDNF sample from an adolescent was lost during BDNF measurements. These values were not replaced. Experiment 1 and 2 were run separately, and each was conducted by running separate groups of approximately 16 animals, representatives of different litters. A period of approximately 6–8 months elapsed between Experiment 1 and 2, hence the CPP data for adolescents and adults were analyzed separately.

CPP habituation scores were analyzed via a two-way mixed analysis of variance [ANOVA, between factors: Treatment and Environmental condition, within factor: absolute time spent in each compartment]. This analysis served to inspect potential bias (i.e., innate, pre-conditioning preferences) for the compartments used during the conditioning. The main dependent variable during test sessions was the absolute (s) or percent preference for the compartment that was used as CS^+^ during conditioning. Specifically, time spent in the CS^+^ compartment was assessed by an ANOVA that considered Treatment and Environmental condition as between factors. The time spent in the CS^+^ compartment during tests 1, 2, 3 and 4 was the repeated measure. Differential CPP scores (i.e., time spent in the CS^+^ compartment at each test session – time spent in the CS^+^ during habituation) were also calculated, yet they are reported as supplementary data only.

We also analyzed the absolute or percent time spent in the compartment paired with ethanol (averaged across the four tests) as a function of Rearing and Treatment, yet including the time spent in each compartment during the pre-conditioning as a co-variate. The aim of conducting these analyses of Covariance (ANCOVA) was to control the possibility that the measure of time spent in the CS^+^ compartment was confounded with any pre-conditioning effects. Furthermore, we compared the time spent in the CS^+^ compartment (s) to the time spent in the CS^−^ compartment, across days of testing, separately for adolescents and adults. This was assessed by repeated measure (RM) ANOVAs that considered Treatment and Environmental condition as between factors. The time spent in the CS^+^ compartment and the time spent in the CS^−^ compartment during tests 1, 2, 3 and 4 were the repeated measures (i.e., 4 testing days, with 2 preference measures in each). To confirm that animals in a given group expressed ethanol-induced CPP, percent scores on test day 1 (i.e., percent time spent in the CS^+^) were analyzed in adolescent and adults, using a *t*-test for single means against a user-defined constant. The constant represented theoretical chance preference for the CS^+^ (50%). A significant difference yielded by the *t*-test was assumed to reflect ethanol-mediated CPP.

In Experiment 3, BDNF levels were assessed using a factorial ANOVA (Age × Treatment × Environmental condition). In this experiment we also analyzed the body weights recorded prior to every administration of saline or ethanol and prior to the habituation session. This served to assess potential deleterious effects of ethanol or environmental rearing conditions upon maturational processes. Because of obvious developmental differences, body weights were analyzed separately in adolescent and adult mice via a Treatment × Environmental condition × Session RM ANOVA.

The loci of the significant effects were analyzed using Newman-Keuls’ *post hoc* test. The alpha level was 0.05 for all analyses, and effect sizes are reported via Cohen’s partial eta squared (η²p).

### Data availability statement

The datasets generated during and/or analyzed during the current study are available from the corresponding author on reasonable request.

## Results

### Habituation, pre-conditioning, CPP scores

The analysis of habituation scores revealed no significant main effect or significant interaction, neither in adolescents nor in adults. In other words, the animals exhibited similar baseline preference for the compartments used during conditioning. Mean and SEM time spent (s) in the mesh or rod compartments, in SC and EE adolescent and adult mice, can be found in Table 1.

**Table 1:** Time spent (s) during the habituation phase of the conditioned place preference procedure, in the mesh or rod compartments, in adolescent and adult mice as a function of rearing condition (standard [control] conditions and environmental enrichment; SC and EE groups, respectively) and treatment during CPP training (vehicle or ethanol).

	Adolescents				Adults			
	SC-vehicle	SC-ethanol	EE-vehicle	EE-ethanol	SC-vehicle	SC-ethanol	EE-vehicle	EE-ethanol
Mesh compartment	315.42 ± 32.27	356.41 ± 18.18	314.82 ± 46.19	266.61 ± 47.46	314.94 ± 17.21	356.90 ± 24.96	321.39 ± 23.25	308.29 ± 28.29
Rod compartment	401.32 ± 46.88	367.84 ± 18.70	347.08 ± 45.97	387.05 ± 75.88	340.47 ± 27.87	353.60 ± 22.31	337.72 ± 23.60	344.34 ± 38.35

The data are presented as mean ± SEM.

### Test, post-conditioning, CPP scores

#### Adolescents

**Figure 2:**
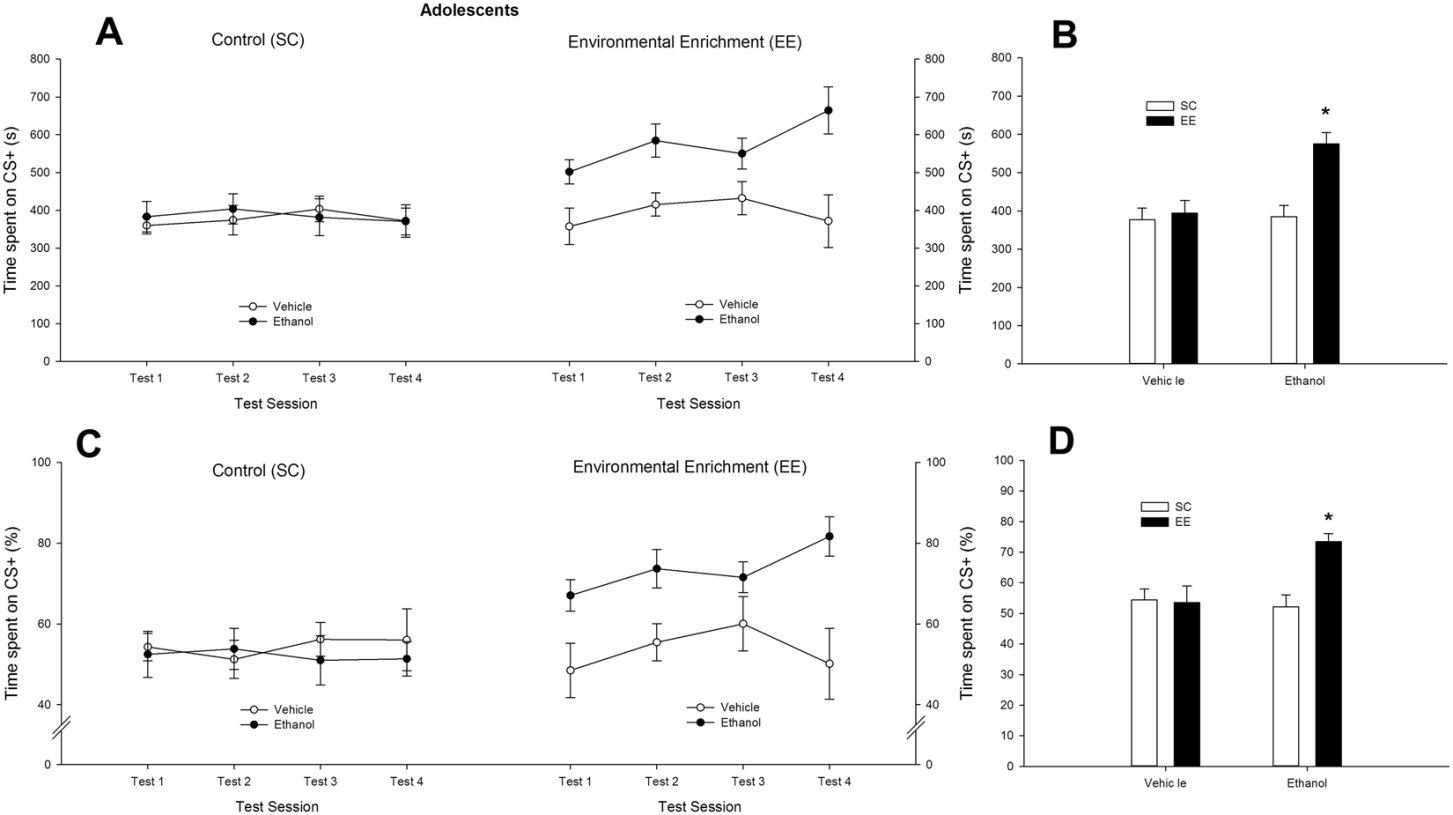
Ethanol-induced conditioned place preference (CPP) in adolescent male Swiss mice, at test sessions 1, 2, 3 and 4. The line graphs depict time spent (in seconds or in percent preference, sections (**A**) and (**C**), respectively) in the chamber associated with ethanol’s effects (conditioned stimulus [CS^+^]), as a function of rearing condition (standard [control] conditions and environmental enrichment; SC and EE groups, respectively) and treatment during CPP training (vehicle or 2.0 g/kg ethanol). The small bar graphs (**B** and **D**) depict absolute (s) and percent time spent in the CS^+^ collapsed across test sessions. Animals that were given ethanol and were reared under EE conditions spent significantly more time in the chamber associated with ethanol than the rest of the animals that were treated with vehicle or ethanol, an effect that was statistically similar across test sessions. This significant effect is indicated by the asterisk sign. Values express mean ± SEM.

The ANOVAs on the absolute or percent time spent in the compartment paired with ethanol during tests 1 to 4 yielded similar results, with significant main effects of Environmental condition and Treatment (absolute time: *F*_1,33_ = 11.48, η²p = 0.26 and *F*_1,33_ = 9.50, η²p = 0.22, respectively; *p* < 0.005; percent time: *F*_1,33_ = 6.43, η²p = 0.17 and *F*_1,33_ = 4.82, η²p = 0.13, respectively; *p* < 0.05) and a significant Environmental condition x Treatment interaction (*F*_1,33_ = 7.62, *p* < 0.001, η²p = 0.19). As shown in Fig. 2, and confirmed by the *post-hocs*, ethanol-treated EE mice displayed a higher absolute and percent time spent in the side paired with ethanol (CS^+^ compartment) compared with EE counterparts that were given vehicle or compared to SC mice given ethanol or vehicle. None of the latter three groups significantly differed between each other and this CPP pattern was similarly expressed in trials 1 to 4.

These results indicate that, among adolescent mice, ethanol-induced CPP was observed only after exposure to EE conditions. The analysis of differential (post-conditioning – pre-conditioning time spent on the CS^+^) CPP scores did not reveal significant main effects or significant interactions. These analysis and scores are provided as Supplementary Fig. 1.

The ANCOVAs that analyzed absolute or percent time spent in the CS^+^ compartment, including time spent in each compartment during the pre-conditioning as a covariate, replicated the effects reported earlier. The ANCOVAs for the absolute and percent scores yielded significant main effects of Rearing (*F*_1,31_ = 9.61, η²p = 0.24 and *F*_1,31_ = 5.22, η²p = 0.22, *p* < 0.05, respectively) and Treatment (*F*_1,31_ = 9.44, η²p = 0.23 and *F*_1,33_ = 9.50, η²p = 0.22, *p* < 0.05, respectively), and significant Rearing x Treatment interactions (*F*_1,31_ = 8.38, η²p = 0.21 and *F*_1,33_ = 7.67, η²p = 0.20, *p* < 0.05, respectively).

Further evidence of the emergence of ethanol-induced conditioning after exposure to EE conditions was provided by the RM ANOVA that compared the time spent in the CS^+^ compartment with the time spent in the CS^−^ compartment. The analysis yielded significant main effects of Rearing and Compartment (*F*_1,31_ = 5.76, η²p = 0.15 and *F*_1,33_ = 17.08, η²p = 0.34, *p* < 0.05, respectively), and significant Rearing x Compartment (*F*_1,33_ = 7.18, η²p = 0.18, *p* < 0.05) and Treatment x Compartment interactions (*F*_1,33_ = 6.72, η²p = 0.17, *p* < 0.05). The interaction between Rearing, Treatment and Compartment also achieved significance (*F*_1,33_ = 9.01, η²p = 0.21, *p* < 0.005). The *post-hoc* tests indicated similar time spent in either compartment in EE or SC animals treated with vehicle or in SC animals treated with ethanol during the CPP procedure. In contrast, EE animals treated with ethanol during the CPP procedure spent significantly more time in the CS^+^ than in CS^−^ compartment. Time spent in the CS^+^ (s) is depicted in Fig. 2, whereas time spent in the CS^−^ in tests 1, 2, 3 and 4 (mean ± SEM) was as follows: 308.37 ± 29.77, 355.81 ± 38.40, 312.47 ± 29.53 and 307.37 ± 29.77, respectively (SC-Vehicle group); 355.32 ± 50.05, 347.11 ± 40.34, 361.78 ± 43.59 and 348.77 ± 32.12, respectively (SC-Ethanol group); 390.20 ± 57.85, 338.92 ± 38.43, 304.87 ± 55.88 and 395.29 ± 82.23, respectively (EE-Vehicle group); and 264.25 ± 29.79, 206.72 ± 38.43, 214.20 ± 27.99 and 134.45 ± 31.79, respectively (EE-Ethanol group).

The *t*-test against a user defined constant revealed that, on test day 1, the mean percent preference for the CS + compartment in EE, but not in SC, adolescents treated with ethanol was significantly higher than 50% (*t*_8_ = 4.38, p < 0.005). The *t*-tests for saline-treated EE and saline-treated SC groups were not significant.

#### Adults

**Figure 3:**
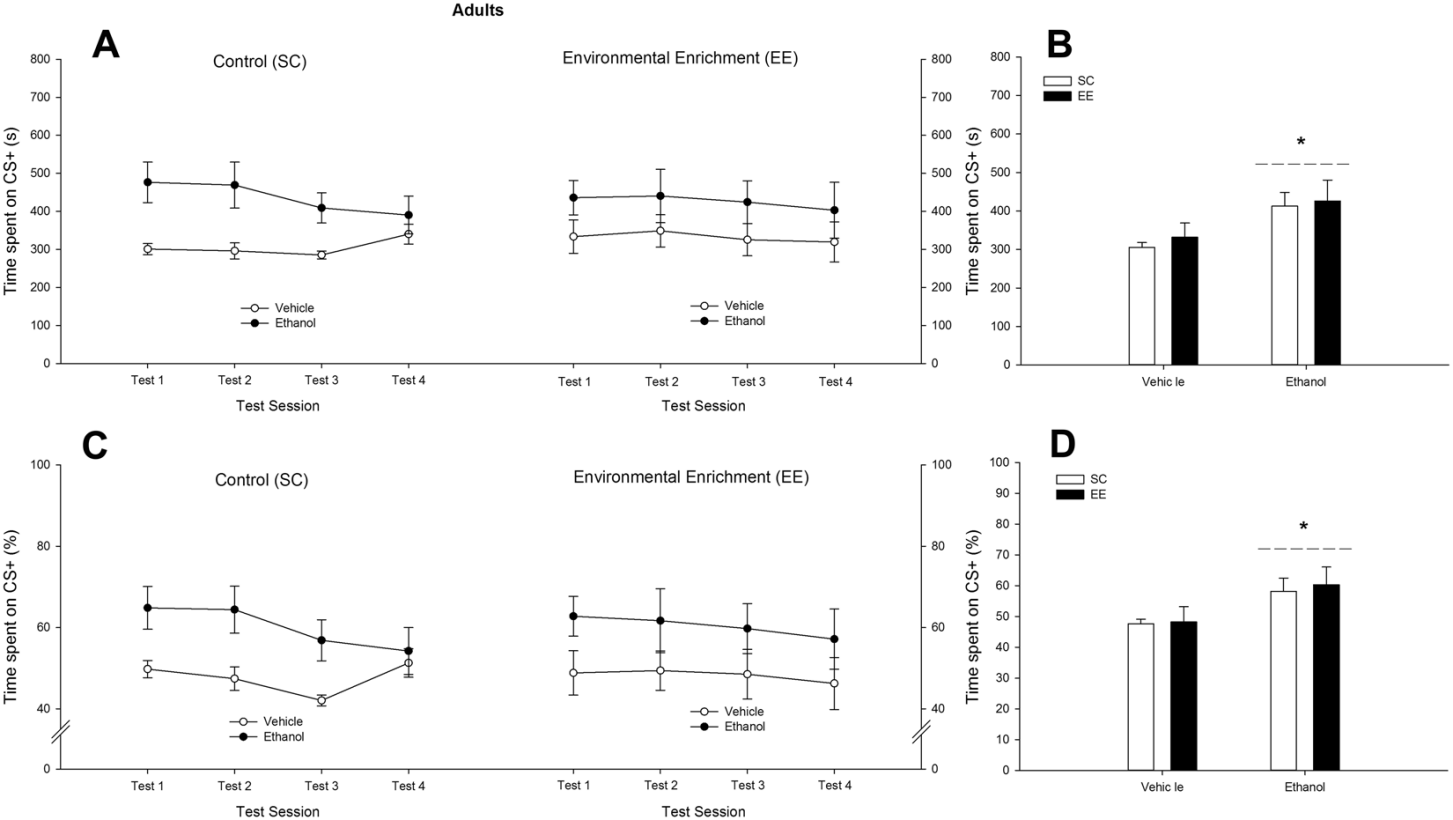
Ethanol-induced conditioned place preference (CPP) of adult male Swiss mice, at test sessions 1, 2, 3 and 4. The line graphs depict time spent (in seconds or in percent preference, sections (**A**) and (**C**), respectively) in the chamber associated with ethanol’s effects (conditioned stimulus [CS^+^]), as a function of rearing condition (standard [control] conditions and environmental enrichment; SC and EE groups, respectively) and treatment during CPP training (vehicle or 2.0 g/kg ethanol). The small bar graphs (**B** and **D**) depict absolute (s) and percent time spent in the CS^+^ collapsed across test sessions. Animals that were given ethanol spent significantly more time in the chamber associated with ethanol than animals that were treated with vehicle, an effect that was statistically similar across test sessions and in EE and SC subjects. This significant effect is indicated by the asterisk sign. Values express mean ± SEM.

The ANOVAs on the absolute or percent time spent in the CS^+^ revealed a significant main effect of Treatment (absolute time: *F*_1,34_ = 6.86, *p* < 0.05, η²p = 0.16; percent time: *F*_1,34_ = 6.38, *p* < 0.05, η²p = 0.16). No significant main effect or significant interaction involving Environmental condition or Testing session was observed. These results, indicative of ethanol-induced CPP which was insensitive to rearing conditions, have been depicted in Fig. 3. Visual inspection of differential CPP scores (see Supplementary Fig. 2) seemed to indicate greater ethanol-induced CPP in EE than in SC adults, yet the ANOVA did not yield significant main effects or significant interactions (e.g., Environmental condition x Treatment interaction: *F*_1,34_ = 2.39, *p* = 0.13, η²p = 0.07).

The ANCOVAs that analyzed the absolute or percent time spent in the compartment paired with ethanol (averaged across tests and including the time spent in each compartment during the pre-conditioning as a co-variate) yielded a significant main effect of Treatment (*F*_1,32_ = 6.20, η²p = 0.16 and *F*_1,32_ = 6.43, η²p = 0.17, *p* < 0.05, respectively). The RM ANOVA that compared the time spent in the CS^+^ compartment with the time spent in the CS^−^ compartment. yielded a significant Treatment x Compartment interaction (*F*_1,34_ = 6.32, η²p = 0.16, *p* < 0.05). The *post-hoc* tests revealed that the adults treated with ethanol during the CPP spent significantly more time at test in the CS^+^ compartment than in the CS^−^ compartment. This was not observed in vehicle-treated controls, which spent similar time in the CS^+^ and in the CS^−^ compartment. Time spent in the CS^+^ (s) is depicted in Fig. 3, whereas time spent in the CS^−^ in tests 1, 2, 3 and 4 (mean ± SEM) was as follows: 307.70 ± 20.79, 331.30 ± 25.30, 395.20 ± 17.50 and 323.00 ± 25.24, respectively (SC-Vehicle group); 248.78 ± 36.58, 245.78 ± 40.05, 274.44 ± 47.15 and 322.13 ± 40.01, respectively (SC-Ethanol group); 349.40 ± 42.27, 350.90 ± 36.37, 362.10 ± 57.52 and 379.30 ± 57.63, respectively (EE-Vehicle group); 250.90 ± 34.71, 260.30 ± 57.59, 272.00 ± 42.71 and 276.20 ± 57.30, respectively (EE-Ethanol group).

The *t*-test for a single mean against a user-defined constant of 50% revealed that, on test day 1, ethanol-treated mice, both EE and SC, exhibited significantly more than 50% of the test in the CS + chamber (*t*_9_ = 2.61, p < 0.05 and *t*_8_ = 2.82, p < 0.05, respectively). The *t*-tests for saline-treated EE and saline-treated SC groups were not significant.

#### BDNF levels

**Figure 4:**
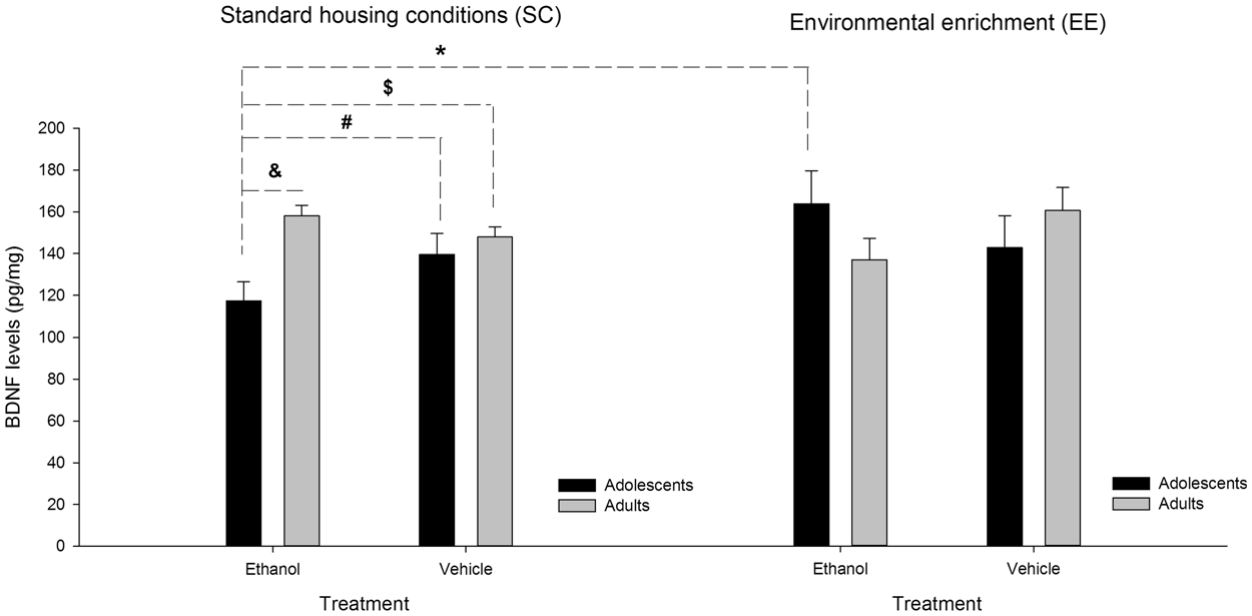
Brain-derived neurotrophic factor (BDNF) levels in prefrontal cortex of adolescent and adult male Swiss mice as a function of rearing condition (standard [control] conditions and environmental enrichment) and treatment (4, every-other-day administrations of 0.0 [vehicle control] or 2.0 g/kg ethanol). The adolescent group treated with ethanol and exposed to standard (control) rearing conditions (first black bar on the left) exhibited significantly less BDNF levels than (**A**) any of the other groups (adolescents or adults) reared under standard housing conditions and treated with vehicle [these significant differences are indicated by the pound (#) and currency ($) signs, respectively], (**B**) the adults treated with ethanol and exposed to standard (control) rearing conditions [a significant difference indicated by the ampersand (&) sign] and **C**) adolescents reared under environmental enrichment conditions and treated with ethanol. The latter significant difference is are indicated by the asterisk (*) sign. Values express mean ± SEM.

The ANOVA for these scores (which are depicted in Fig. 4) indicated a significant three-way Age × Environmental condition × Treatment interaction (*F*_1,69_ = 6.80, *p* < 0.005, η²p = 0.09). Two-way ANOVAs were conducted to better understand the three-way interaction. Separate Treatment x Age ANOVAs for each housing condition were first conducted. The ANOVA for SC mice revealed a significant main effect of Age and a significant Age x Treatment interaction (*F*_1,37_ = 11.69, η²p = 0.24 and *F*_1,37_ = 5.05, η²p = 0.12 respectively; *p* < 0.05). *Post-hoc* comparisons indicated that BDNF levels were significantly lower in SC adolescent mice given ethanol, compared to SC adolescent mice that received vehicle, and also compared with SC adults given either treatment. The ANOVA for EE mice revealed a lack of significant main effects or significant interactions. We subsequently conducted separate Environmental condition x Age ANOVAs for each treatment condition. The ANOVA for vehicle-treated animals did not yield significant main effects or significant interactions. On the other hand, the ANOVA for ethanol-treated animals yielded a significant Age x Environmental condition interaction (*F*_1,35_ = 10.03, η²p = 0.22, *p* < 0.005). As revealed by *post hoc* comparisons, BDNF levels were significantly lower in ethanol-treated adolescent mice that were reared under standard housing conditions compared with adult counterparts that were exposed to similar treatments (i.e., SC-ethanol adult group). The reducing effect of ethanol treatment during adolescence was not observed in EE animals (i.e., among ethanol-treated adolescents, BDNF levels were significantly higher in the EE than in the SC group).

**Figure 5:**
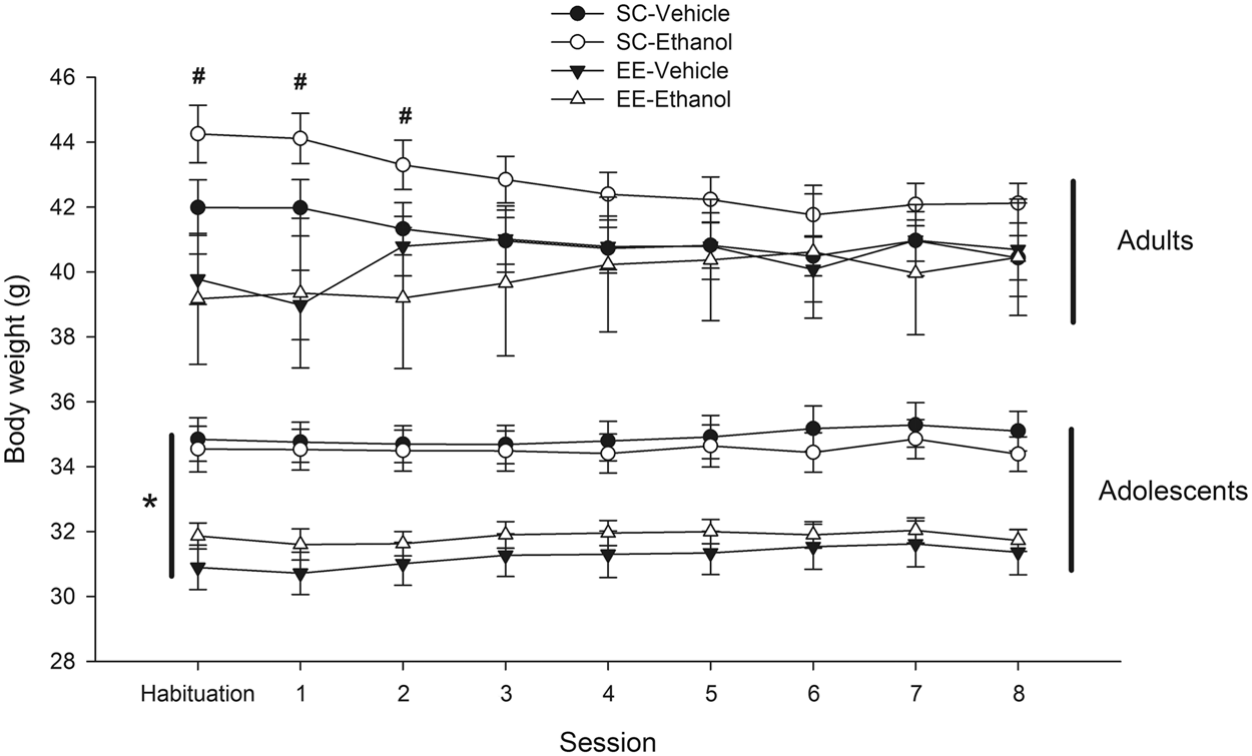
Body weight (g, mean ± SEM) of male Swiss mice, prior to every administration of saline or ethanol (sessions 1 to 8) and prior to the habituation session, as a function of Age (adolescents or adults), Environmental condition (environmental enriched [EE] or standard housing conditions [SC]) and Treatment (4, every-other-day administrations of 0.0 [vehicle control] or 2.0 g/kg ethanol). The asterisk sign indicates that, throughout days, body weight was significantly lower in EE adolescent animals than in SC counterparts. The pound sign indicates a significant difference between EE adults given ethanol and vehicle, SC adults, during habituation and sessions 1 and 2.

Throughout habituation and treatment days, body weight was lower in EE adolescent animals than in SC counterparts. The ANOVA indicated a significant main effect of Environmental condition (*F*_1,33_ = 18.88, η²p = 0.36, *p* < 0.001). The ANOVA for adult mice indicated a significant Environmental condition × Day interaction (*F*_8,296_ = 18.86, η²p = 0.34, *p* < 0.001). The three-way Environmental condition × Day × Treatment interaction was also significant (*F*_8,296_ = 2.31, η²p = 0.06, *p* < 0.05). The *post hoc* tests indicated that adults that were reared under EE conditions and treated with ethanol exhibited a reduction of body weight compared with SC controls treated with ethanol, although only during the first three measurements. Body weight across habituation and conditioning sessions is depicted in Fig. 5.

## Discussion

In the present study, housing conditions impacted CPP by ethanol only in the adolescents. This is, an enriched housing (i.e., a home cage filled with rotating sets of novel objects) promoted the emergence of CPP by ethanol in the adolescents, but did not significantly alter the expression of CPP in the mature animals. Furthermore, adolescents reared under SC conditions that were exposed to ethanol treatment exhibited lower levels of BDNF in the PFC than adults similarly exposed to ethanol and SC rearing, and than adolescents given vehicle under control conditions. This ethanol-induced reduction in BDNF levels was not observed in adolescents reared under EE conditions. We will now discuss in depth each of these key findings.

Experimentation with ethanol begins between 8 and 12 years of age^33^. By 14–15 years of age, regular use is widespread and escalates up to 18–24 years of age, when maximum levels of problematic ethanol consumption are reached. Ethanol consumption exhibits a sudden drop during the transition into adulthood^34^. Consistent with epidemiological studies, animal models with mice^35^ and rats^36, 37^ have found greater ethanol intake in adolescent subjects than in adult subjects. Ontogenetic differences in the sensitivity to ethanol’s motivational effects may underlie the greater predisposition to ethanol consumption in youths. Rat studies indicate that adolescents appear to be more sensitive than adults to ethanol’s appetitive effects^25^. Adolescent mice^35^ and rats^38^ are less sensitive to the aversive effects of higher ethanol doses. Ethanol-induced behavioral stimulation is also greater in female adolescent mice^39^ and rats^40^, and adolescents from these species exhibit less ethanol-induced sedation^4, 41^ than adult counterparts.

Based on this background, it was possible that adolescent Swiss mice would exhibit, regardless the characteristics of their rearing environment, greater ethanol-induced CPP than their adult counterparts. This expectation, however, was not met. Among mice reared under standard animal facility conditions (i.e., SC groups), ethanol resulted in CPP in adult but not in adolescent mice. The lack of ethanol-induced CPP in adolescent mice reared under SC housing is less surprising when considering that CPP studies in adolescent mice have yielded inconsistent results, that are unlike the reliable CPP observed in adult mice. Roger-Sanchez, *et al*.^42^ reported ethanol-induced CPP in early and late adolescent females given 2.5 g/kg ethanol and early adolescent males given 1.25 or 2.5 g/kg ethanol. Intriguingly, late adolescent males did not exhibit CPP. This elegant study indicated a gradual decrease in the rewarding effects of ethanol with age, but the study lacked a comparison with an adult group. Such comparisons were made in other studies^7, 27^, which suggested that adolescent mice are actually less sensitive than adult mice to ethanol-induced reward. Specifically, in these studies adolescent DBA/2 J mice required a higher dose of ethanol than adults to exhibit ethanol-induced CPP [4.0 *vs*. 2.0 g/kg, respectively^27^], whereas the adolescent mice in Song, *et al*.^7^ only exhibited CPP after stress exposure.

The genetics of the mice used across the studies is another potential reason for the discrepancies observed in the literature. Roger-Sanchez *et al*.^42^ employed OF1 mice, versus the DBA/2 J used by Dickinson *et al*.^27^, versus the Swiss used in the current study. DBA/2 J is an inbred strain particularly sensitive to the development of behavioral sensitization^43^. Adolescent and adult DBA/2 J mice do not differ in ethanol consumption [as measured through the drinking in the dark procedure^3^] yet adolescents of this strain are, when compared to adults, less sensitive to ethanol-induced sensitization^44^. In an ethanol-induced CPP study^45^ (dose: 2.0 g/kg), adult DBA/2 J mice spent around 60% of the test on the CS^+^, which is similar to our data in Swiss mice. DBA/2 J mice, however, exhibited a negative correlation between ethanol intake and ethanol-induced CPP^45^, whereas pre-exposure to ethanol potentiates ethanol conditioning in the Swiss mice^46^. Adolescent Swiss mice also show, when compared to adult counterparts, lower levels of sensitization to ethanol^6^ yet after the sensitization ethanol intake escalates more pronounced in the adolescents than in the adults^47^, Studies with the outbred strain OF1 have shown that pre-exposure to ethanol or to stress during adolescence enhances subsequent cocaine-induced CPP^48^ and ethanol self-administration^49^, respectively. These studies, however, did not have an adult control group for comparison. In summary, the literature on ethanol-induced CPP across age in mice provides a seemingly contradictory pattern, which to some extent may be attributable to procedural differences across studies, including ethanol dose, duration of conditioning and testing, the presence of other stressors, the intensity and duration of CS exposure and the genetics of the animals tested. Although this heterogeneity makes impossible to draw definitive conclusions, the present study supports the possibility that, in mice, adolescents exhibit a blunted response to the rewarding effects of ethanol, as measured via CPP.

An important finding of the present study is the robust CPP found in adolescents reared under EE, but not under SC, conditions. This suggests that exposure to, at least certain types, of environmental enrichment may act as a vulnerability factor during adolescence, facilitating seeking of ethanol or ethanol-related cues. This is consistent with a recent study^18^ reporting enhanced ethanol intake in male rats that, during adolescence, had been exposed to the structural enrichment employed in our work. Berardo *et al*.^18^ also found that EE increased exploratory and risk taking behaviours, and linked this phenotype to the increased ethanol intake, found after EE. On the other hand, EE did not significantly modulate ethanol-induced CPP in adult animals. An important caveat for these conclusions, and a limitation of our work, is that our animals were exposed to EE only for 15 days before the CPP procedure started. Studies showing a protective effect of EE on cocaine consumption exposed animals to EE for longer periods of time (e.g., 30 days^50^) or kept animals in EE from weaning to adulthood^51, 52^. It is thus possible that the lack of effect of EE in ethanol-induced CPP at adulthood was due to short time of exposure to EE. Another reason for this lack of an effect could be that we exposed the adult group to EE only during adulthood and not during adolescence, when the brain is relatively more plastic.

It is also interesting to discuss the CPP pattern found in the present study with that reported by Carrara-Nascimento, *et al*.^6^. In the latter study ethanol pre-exposed adolescent mice and vehicle pre-exposed adult mice exhibited reliable ethanol-induced CPP. This conditioned preference was not exhibited by vehicle pre-exposed adolescent mice. Substantial ethanol pre-exposure has been long known to facilitate expression of ethanol-induced CPP in species or strains otherwise refractory to this learning [e.g., adult rats^53, 54^]. The present study pinpoints EE as another factor that can promote, at least when given during adolescence, the emergence of CPP by ethanol. Important new information was that the CPP patterns reported in adolescent and adults were fairly similar across the testing sessions.

The most important implication of the BDNF results (Experiment 3) is that there appears to be an age-related difference in the effect of ethanol treatment on BDNF levels at PFC, which does not emerge if subjects are exposed to EE. As described, the animals were kept under SC or EE conditions before and during ethanol exposure (i.e., throughout the course of the experiments). It is tempting, then, to speculate that EE conditions facilitated the expression of ethanol-induced CPP in the adolescents of Experiment 1 by protecting them of the effects of ethanol on BDNF levels at PFC. This could suggest that BDNF was a possible mediator of the effects observed. This conclusion, however, has to be greatly tempered. First, BDNF was not measured in animals that underwent CPP, and thus cannot be subjected to correlational analysis verifying mediation. Second, there is dissociation between BDNF expression and ethanol-induced CPP across age group, suggesting that changes in BDNF are not necessary for the expression of ethanol-induced CPP.

The effects of EE on BDNF in the PFC have been less studied, and the results have been more controversial than those found in the hippocampus^55^. Chronic maternal separation, a potent stressor, resulted in a decrease and increase in BDNF gene expression in the ventromedial and dorsomedial sections of the PFC, respectively^56^. Voluntary exercise is thought to be a critical component of EE and was included in our study as wheel running. Exercise was shown to increase the low levels of hippocampal BDNF caused by ovariectomy but had no effect on BDNF levels in the PFC^57^. Our data support these findings, indicating that EE during adolescence did not alter overall BDNF levels in the PFC.

Aside from the effect on the subgroup of adolescent mice that were exposed to ethanol under EE, environmental enrichment did not significantly affect BDNF levels in the other groups. These results appear to conflict with the data reported by Rueda, *et al*.^12^, who found a decrease in BDNF levels in the PFC after EE. Critical differences between the present study and Rueda *et al*.^12^ are the length of EE (26 days *vs*. 40 days, respectively), the age of the subjects at the beginning of EE (21 days *vs*. 28 days, respectively). As mentioned above, discrepancies commonly exist between studies that assess BDNF after EE. Many studies indicated a facilitative effect of EE on hippocampal BDNF levels or gene expression^58^, but others reported no changes after comparable treatment^59^.

The results found in this study have to be met in the context of important, limitations. The PFC is divided in several sub-regions with different functions^60^. Therefore, measuring BDNF levels in this structure as a whole may have resulted in levels not representative of those found in the specific subregions. Also, a single dose of ethanol (2.0 g/kg) was used in the CPP study. It is possible that a different pattern would have emerged had we used additional higher or lower, ethanol doses. The 2.0 g/kg ethanol dose, however, is a standard dose for inducing CPP in mice and has been used in several influential studies that assessed factors influencing the expression of ethanol’s motivational effects^61–64^.

In summary, the present data suggests that, adolescent, but not adult, Swiss mice exhibit a facilitatory effect of EE on ethanol-induced CPP. Moreover, ethanol treatment reduced BDNF levels at PFC in adolescents reared under control (standard housing) conditions. This effect was not observed in SC or EE adults nor in adolescents exposed to EE. These effects add to the plethora of adolescent-specific responses to ethanol^65^ that may put the youth at risk for initiation or escalation of ethanol intake.

## Electronic supplementary material

Supplementary Figures 1 and 2
