# Supplementary material for: Effects of environmental enrichment upon ethanol-induced conditioned place preference and pre-frontal BDNF levels in adolescent and adult mice
Source: Sci Rep. 2017 Aug 17;7:8574. doi: 10.1038/s41598-017-08795-0 (PMC5561235; doi:10.1038/s41598-017-08795-0)
Supplement: Supplementary file 1 — Supplementary Figures 1 and 2 [file 41598_2017_8795_MOESM1_ESM.pdf]

Effects of environmental enrichment upon ethanol-induced conditioned place preference and pre-frontal bdnf levels in adolescent and adult mice. RM Pautassi, AB Suárez, L Barbosa Hoffmann, AV Rueda, M Rae, P Marianno & R Camarini

Supplementary Figure S1. Ethanol-induced conditioned place preference (CPP) in adolescent male Swiss mice, at test sessions 1, 2, 3 and 4. The graphs depict differential CPP scores (i.e., time spent in the CS+ compartment at each test session – time spent in the CS+ during habituation, seconds) as a function of rearing condition (standard [control] conditions and environmental enrichment; SC and EE groups, respectively) and treatment during CPP training (vehicle or 2.0 g/kg ethanol). Values express mean ± SEM. The table presents the ANOVA table of effects. There were no significant main effects or significant interactions.

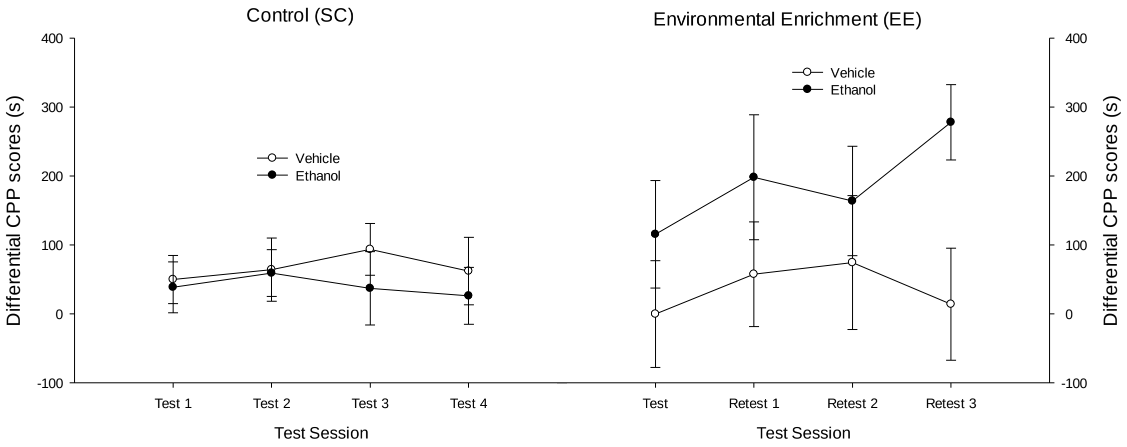

Effect	Repeated Measures Analysis of Variance with Effect Sizes and Powers SS	Degr. of Freedom	MS	F	p	Partial eta-squared	Non-centrality	Observed power (alpha=0,05)
**Intercept**	1021212	1	1021212	9,176745	0,004736	0,217578	9,176745	0,836488
Rearing Conditions	127661	1	127661	1,147182	0,291911	0,033595	1,147182	0,180170
Treatment	144915	1	144915	1,302221	0,262022	0,037963	1,302221	0,198230
Rearing Conditions*Treatment	297782	1	297782	2,675912	0,111381	0,075006	2,675912	0,355256
Error	3672327	33	111283					
M	51687	3	17229	1,450886	0,232673	0,042115	4,352658	0,373921
M*Rearing Conditions	36869	3	12290	1,034929	0,380592	0,030408	3,104786	0,273406
M*Treatment	45420	3	15140	1,274957	0,287201	0,037198	3,824871	0,331545
M*Rearing Conditions*Treatment	45057	3	15019	1,264776	0,290694	0,036912	3,794328	0,329080
Error	1175609	99	11875					

Effects of environmental enrichment upon ethanol-induced conditioned place preference and pre-frontal bdnf levels in adolescent and adult mice. RM Pautassi, AB Suárez, L Barbosa Hoffmann, AV Rueda, M Rae, P Marianno & R Camarini

Supplementary Figure S2. Ethanol-induced conditioned place preference (CPP) in adult male Swiss mice, at test sessions 1, 2, 3 and 4. The graphs depict differential CPP scores (i.e., time spent in the CS+ compartment at each test session – time spent in the CS+ during habituation, seconds) as a function of rearing condition (standard [control] conditions and environmental enrichment; SC and EE groups, respectively) and treatment during CPP training (vehicle or 2.0 g/kg ethanol). Values express mean ± SEM. The table presents the ANOVA table of effects. There were no significant main effects or significant interactions.

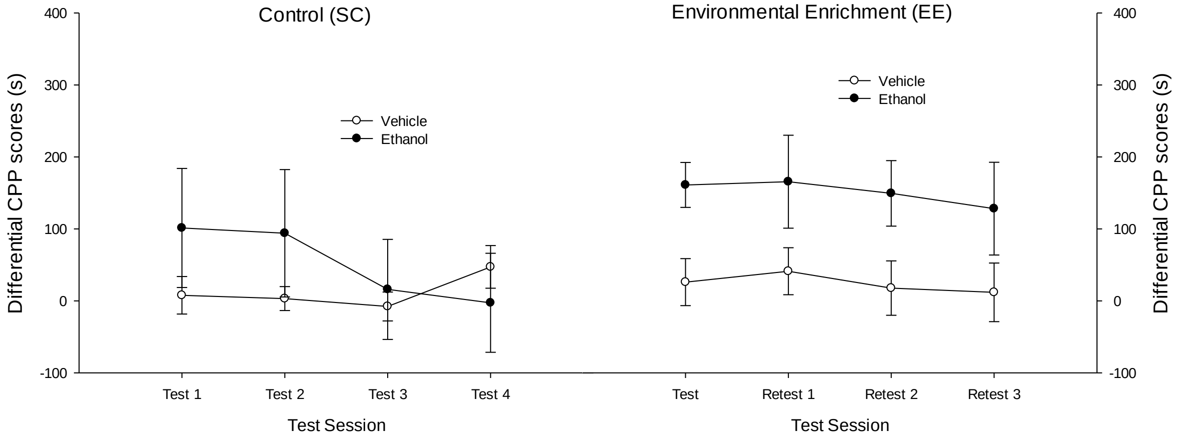

Effect	SS	Degr. of Freedom	MS	F	p	Partial eta-squared	Non-centrality	Observed power (alpha=0,05)
	Repeated Measures Analysis of Variance with Effect Sizes and Powers							
**Intercept**	403991	1	403991,3	7,139505	0,011492	0,173544	7,139505	0,737585
Rearing Conditions	192646	1	192646,3	3,404527	0,073743	0,091019	3,404527	0,433778
Treatment	168572	1	168572,3	2,979081	0,093426	0,080561	2,979081	0,388842
Rearing Conditions*Treatment	135120	1	135120,3	2,387903	0,131536	0,065624	2,387903	0,323658
Error	1923901	34	56585,3					
TESTS	7241	3	2413,6	0,328336	0,804866	0,009565	0,985007	0,111949
TESTS*Rearing Conditions	9229	3	3076,4	0,418494	0,740107	0,012159	1,255482	0,130915
TESTS*Treatment	14954	3	4984,6	0,678078	0,567434	0,019554	2,034235	0,188894
TESTS*Rearing Conditions*Treatment	6792	3	2263,9	0,307970	0,819575	0,008977	0,923910	0,107767
Error	749804	102	7351,0					
